# Bronchiectasis insanity: Doing the same thing over and over again and expecting different results?

**DOI:** 10.12688/f1000research.17295.1

**Published:** 2019-03-15

**Authors:** Mark Metersky, James Chalmers

**Affiliations:** 1 Division of Pulmonary, Critical Care and Sleep Medicine, University of Connecticut School of Medicine, Farmington, USA; 2 Scottish Centre for Respiratory Research, Ninewells Hospital and Medical School, Dundee, UK

**Keywords:** Bronchiectasis, Treatment, Clinical trial

## Abstract

Bronchiectasis is an increasingly common disease with a significant impact on quality of life and morbidity of affected patients. It is also a very heterogeneous disease with numerous different underlying etiologies and presentations. Most treatments for bronchiectasis are based on low-quality evidence; consequently, no treatments have been approved by the US Food and Drug Administration or the European Medicines Agency for the treatment of bronchiectasis. The last several years have seen numerous clinical trials in which the investigational agent, thought to hold great promise, did not demonstrate a clinically or statistically significant benefit. This commentary will review the likely reasons for these disappointing results and a potential approach that may have a greater likelihood of defining evidence-based treatment for bronchiectasis.

Bronchiectasis is an increasingly common disease. In the US, the condition was being actively treated in an estimated 340,000 to 522,000 patients in 2013 ^1– 3^. These same data suggested an annual increase in prevalence of 8% ^1^. When the authors of this commentary were developing an interest in bronchiectasis, it was widely considered an orphan disease ^4^. There were no treatments approved by the US Food and Drug Administration (FDA) or the European Medicines Agency (EMA), and there was limited evidence of interest among pharmaceutical companies in developing therapies that would change that situation. Then, around 10 years ago, it appeared that bronchiectasis was an orphan no longer. Patient registries dedicated to research were initiated, first in the United States and then in Europe and the Asia-Pacific region ^5, 6^. Perhaps, informed by epidemiologic studies revealing that the prevalence of bronchiectasis was much greater than that of cystic fibrosis (CF) (for which there are numerous FDA-approved therapies), pharmaceutical companies started showing interest. Ultimately, after initial study, a series of clinical trials, including phase III clinical trials, were conducted, and there was great hope that the results would usher in a new era of evidence-based high-quality care ^7– 10^. Unfortunately, these studies either failed to meet their primary endpoint or demonstrated inconsistent benefit. Inhaled mannitol failed ^10^. Inhaled aztreonam failed ^7^. Inhaled colistin failed ^11^. Inhaled dry powder ciprofloxacin failed ^8, 9, 12^. Consequently, in 2018, there were still no therapies approved for bronchiectasis by the FDA or EMA. This commentary will explore potential explanations for these failures and discuss a recommended path forward.

## Are we studying some drugs in the wrong disease?

Most therapies that have been studied in patients with bronchiectasis were initially developed and used to treat CF ^13^. At some level, this makes sense; the two conditions share the commonality of impaired airway local host defenses and resulting chronic airway infection. However, bronchiectasis is not CF.

DNase (dornase alpha) is a mucolytic, as DNA released extracellularly from polymorphonuclear leukocytes imparts much of the viscosity of CF sputum ^14– 16^. DNase slows the loss of pulmonary function in CF and is a mainstay of treatment. However, in bronchiectasis, DNase resulted in increased risk of pulmonary exacerbations compared with placebo and no benefit in any other parameters ^16^.

Hypertonic saline nebulization, commonly used as an adjunct for airway clearance therapy in CF, acts as an osmotic agent, drawing water into the airways. However, in a 12-month randomized controlled trial, 6% saline was no better than isotonic saline with respect to effect on quality of life (QoL) and pulmonary function ^17^. Mannitol, in addition to functioning as an osmotic agent, enhances ciliary beat frequency, theoretically resulting in easier sputum clearance ^10, 18^. Because it persists in the airway for longer than hypertonic saline, it was hoped that it would be more effective in improving mucociliary and cough clearance. Indeed, it resulted in improved pulmonary function in patients with CF and was approved for use in the UK and Australia. In 2014, a phase III trial of inhaled mannitol in bronchiectasis was reported ^10^. There were no significant improvements in the primary endpoint: exacerbation rate with inhaled mannitol; consequently, it is not approved anywhere for use in bronchiectasis. Statistically significant improvements were seen in time to first exacerbation and St George’s Respiratory Questionnaire (SGRQ), although the mean improvement in SGRQ was not greater than the minimally important difference ^10^.

The underlying defect in CF is abnormally viscous and tenacious mucus, which impairs cough and ciliary clearance ^19^. The fundamental epithelial and ciliary defects are understood, and drugs such as DNase and mannitol have been specifically formulated on the basis of this knowledge. In contrast, there has been minimal research into cilia or epithelial function in bronchiectasis, and mucus characteristics are poorly understood ^20^. However, in most patients with bronchiectasis, the mucus is normal, and most patients seem to have less difficulty clearing it unless they develop advanced disease that impairs cough efficacy. Therefore, it is not surprising (in retrospect) that therapies aimed at reducing mucus viscosity and increasing mucus water content might not be useful in many patients with bronchiectasis despite the demonstrated benefit in CF. Indeed, one study demonstrated that, in contrast to CF, DNase worsened mucus transportability in bronchiectasis and therefore may exacerbate airway obstruction ^21^. Ongoing or planned studies of agents targeting mucociliary clearance include studies of *N*-acetylcysteine and an epithelial sodium channel (ENaC) inhibitor, VX-371 ^20^.

## Are we studying the right drugs in the right patients but giving the drugs the wrong way?

Worldwide, inhaled antibiotics are frequently used “off-label” in bronchiectasis. These include tobramycin, colistin, gentamicin, aztreonam, and vancomycin. Expert opinion, early-phase studies, and even some phase III trials suggest that these agents result in significant improvement in QoL and reduce frequency of exacerbations in some patients with bronchiectasis ^22, 23^. In contrast to the practice in Europe, where inhaled antibiotics are given on a continuous schedule, in the US, most patients with bronchiectasis are given inhaled antibiotics on a 28-days-on/28-days-off schedule ^23^. This schedule originated with the phase III study of inhaled tobramycin for CF, published in 1999 ^24^. The reported rationale for this schedule was that the off periods would “allow susceptible pathogens to repopulate the airways in patients with cystic fibrosis”, thereby limiting the development of resistance ^6^. However, it is not clear that this rationale makes sense for repeated cycles, and a recent systematic review in bronchiectasis confirms that the hypothesis that this approach would limit resistance development has never been tested ^25^. In addition to the continuous use of a single inhaled antibiotic, there is evidence in CF suggesting improved outcomes with the use of continuous alternating inhaled antibiotics, specifically patients did better with inhaled tobramycin alternating with inhaled aztreonam (each for 28 days), compared with inhaled tobramycin for 28 days alternating with 28 days of placebo ^26^.

Another rationale for the 28-day on/off schedule is that the 1999 CF tobramycin study demonstrated that the achievable increase in FEV _1_ (forced expiratory volume in 1 second) from inhaled antibiotics occurred at 28 days and further increases were not seen with a longer course ^24^. However, there are reasons to question the wisdom of this practice. As mentioned above, FEV _1_ improvement is not an appropriate outcome for bronchiectasis, as FEV _1_ is generally impacted to a lesser extent compared with CF by exacerbations and responds minimally to antibiotic treatment for bronchiectasis ^11^. A potential indication that the 28-day cycle is not the optimum schedule for bronchiectasis comes from the recently reported RESPIRE 1 trial, in which a 14-day on/off cycle of inhaled ciprofloxacin dry powder improved time to first exacerbation and exacerbation frequency compared with placebo but a 28-day on/off cycle did not ^8, 9^. This observation is certainly not definitive, as the identically structured RESPIRE II trial found no statistically significant improvements in either outcome in either the 14- or 28-day groups, although the trends to improvement were greater in the 28-day on/off patients ^8, 9^. However, it is not unusual for patients on the 28-day on/off regimen to report increasing cough and sputum production near the end of their 28-day off cycle and bacterial density certainly increases by the end of the off cycle ^7^.

## Are we studying the right drugs in the right patients but using the wrong outcomes?

Most of the large clinical trials in bronchiectasis have used frequency of exacerbation or time to exacerbation as the primary outcome ^8, 9, 11^. There is no doubt that exacerbations are an important cause of morbidity and, to a lesser extent, mortality in patients with bronchiectasis ^27, 28^. But for many patients, the daily burden of cough and sputum is perceived as an equal or greater concern ^29^. Furthermore, in most patients, exacerbations are comparatively uncommon events, occurring once or twice a year ^6, 30^. This creates several problems. First, since there needs to be a high-enough baseline exacerbation rate to allow detection of a drug effect, most trials enroll patients with only two or more exacerbations in the prior year. So we have been studying a non-representative, relatively low-prevalence patient subgroup (albeit one with a markedly worse prognosis) ^31^. Furthermore, we have frequently used a primary endpoint that may have less significance to the patient than to us investigators. The analysis of exacerbations is also complicated by the multiple different methods of analysis, including the time to first exacerbation, which is a relatively “clean” endpoint but which ignores all events following the first exacerbation, therefore potentially reducing the complex impact of bronchiectasis on a patient life over decades to a single point in time. Exacerbation frequency is more holistic but is complicated by the challenge of separating whether multiple antibiotic courses within a short time represent distinct exacerbations or a single worsening of symptoms. Exacerbations are also inter-dependent events, as patients are much more likely to have another exacerbation soon after they have the first ^28^. Commonly used methods of analysis for exacerbations such as negative binomial models fail to account for this.

A common joke among bronchiectasis specialists is that the best way to prevent exacerbations among our patients is to consider enrolling them in a clinical trial. Most studies have shown lower-than-expected exacerbation rates in the placebo group, limiting the potential for positive results ^8, 9^. Although the closer medical attention paid to clinical trial participants might be partly responsible, regression to the mean is likely a major factor. Consider a patient who for many years has had zero or one exacerbation a year but who one year has an additional exacerbation after being exposed to her sick grandchild. She qualifies for the trial by virtue of her two exacerbations, but there is no reason to expect that she and patients like her are at high ongoing risk of exacerbation during the trial.

An ongoing phase III clinical trial of an agent that prevents release of neutrophil proteases is also using time to exacerbation as the primary outcome ^32^. Proteases contribute to progressive airway damage and increase mucus secretion in patients with bronchiectasis ^33^, so one could theorize that such a drug could be beneficial in decreasing cough, improving QoL, and preventing progression of disease without decreasing the frequency of exacerbations, which of course are usually caused by infectious agents. However, the rate of lung function loss in bronchiectasis is slow; any study that had lung function as a primary endpoint would likely need to continue for years to have a chance of showing benefit.

The issues noted above make it difficult to demonstrate improved exacerbation rates or stability of lung function in bronchiectasis clinical trials. Yet there are not any clearly better options for primary endpoints. Sputum volume would seem to have face validity but for several reasons is not optimum. Some patients with bronchiectasis hesitate to expectorate their sputum. Some believe a higher volume of sputum production represents clinical improvement as the sputum is easier to bring up. Others would be thrilled to produce less sputum, whereas some patients are worried when they produce less sputum believing that a lower quantity of mucus reflects increasing difficulty in sputum clearance and greater sputum retention. Given the heterogeneity of bronchiectasis, perhaps each of these perceptions is “correct” in some patients ^34^.

QoL, measured with a bronchiectasis-specific instrument, was used as the primary endpoint in the phase III inhaled aztreonam trials which demonstrated no improvement in QoL in AIRBX1 and a small statistically significant improvement which did not exceed the minimum clinically important difference in AIRBX2 ^7^. The failure may have been partly related to an increased rate of adverse events due to the therapy.

The three trials ^35– 37^ demonstrating improved exacerbation rates resulting from chronic low-dose macrolide therapy demonstrated limited and inconsistent benefit with respect to QoL, suggesting an important disconnect between exacerbations and symptom improvements with therapy. The recent RESPIRE trials ^8, 9^ used two QoL tools: the Quality of Life-Bronchiectasis respiratory symptom score (QoL-B) and the SGRQ. These trials again demonstrated this disconnect but also showed conflicting results with the two tools, suggesting that they may measure subtly different aspects of the disease. Some regulatory agencies have been unenthusiastic about accepting QoL as a primary endpoint for bronchiectasis trials. Although QoL and symptoms are an enormously important outcome for patients with bronchiectasis and in an ideal world would be the primary endpoint in trials, there is insufficient confidence in the existing tools to recommend using one as the primary endpoint in a future trial. Other potentially useful QoL tools for bronchiectasis trials include the Leicester Cough Questionnaire, which has been used in positive trials of cough-related illness ^38^, and the Bronchiectasis Health Questionnaire, with which there is less clinical experience than the QoL-B but which has the potential advantages of being briefer and yielding a single score ^39^. Finally, measurement of cough frequency is a potential objective outcome that may correlate well with QoL in patients with bronchiectasis ^40^.

## Are we studying the right drugs but not identifying the right subpopulations of patients with bronchiectasis who would benefit from them?

Bronchiectasis is an extremely heterogeneous disease ^33^. Patients differ greatly in terms of underlying etiology, severity of disease, frequency of exacerbations, and prognosis ^41^. Underlying the relatively subtle clinical differences evident in daily practice are multiple complex endotypes (biological processes which link to clinical manifestations or treatment response) ^20, 33^. The era of personalized medicine in CF and asthma has led to the use of therapies targeted on the basis of genotype and biomarkers. Bronchiectasis is substantially more heterogeneous than these two conditions, but for the most part, other than requiring evidence of exacerbations during the prior year and chronic sputum production (and an appropriate target organism for inhaled antibiotic trials), most trials have not distinguished between different phenotypes of patients.

Two phase II studies of oral neutrophil elastase inhibitors (BAY 85-8501 and AZD9668) have been conducted during the last 5 years without clear evidence of beneficial effects but did not include patients on the basis of elevated levels of neutrophil elastase or other neutrophil biomarkers ^42, 43^. Twenty to thirty percent of patients with bronchiectasis appear to have predominantly eosinophilic- or non-neutrophil-dominant inflammation ^44^. The aim of inhaled antibiotics is to reduce bacterial load, and high bacterial loads are associated with airway inflammation and future exacerbation risk ^45^. Yet inhaled antibiotic studies enroll patients with positive sputum cultures which may include levels of bacteria not associated with exacerbation risk and not likely to respond to inhaled antibiotics ^8, 9^. Inhaled corticosteroids are widely used in clinical practice but as yet have not been targeted toward eosinophilic inflammation where response has been demonstrated in other diseases ^46^. Mucoactive drugs are tested in populations of patients with widely heterogeneous sputum characteristics without establishing whether sputum DNA is elevated (in the case of DNase) or mucus dehydration is present (in the case of mannitol). Simple and easy-to-identify biomarkers could help to identify patients who would be more likely to benefit from specific therapies. It remains to be seen whether specific bronchiectasis etiologies can help predict the presence of these biomarkers, although at this point there is no such evidence. Distinct from the underlying bronchiectasis etiology, the different patient characteristics and biomarkers which link to treatment responses can be called “treatable traits” and this approach has great potential to cut through the complexity of bronchiectasis to perform more targeted and more rational clinical trials ^47^.

## Summary

Bronchiectasis is an extremely heterogeneous condition that exacts a tremendous toll on QoL of many afflicted patients. The initial optimism about the increasing attention being paid to bronchiectasis in the last decade has waned somewhat with each successive trial that did not demonstrate improvement in the primary endpoint. There appear to be multiple reasons for these results, but they include application of principles relevant to CF without adequate consideration of the differences between the two diseases. Another has been incomplete characterization of the phenotypic, genetic, and endotypic variations in patients with bronchiectasis, such that potential therapies are applied to “all comers”, even though many patients might not have characteristics that would predict therapeutic success. Furthermore, determining the appropriate primary endpoint is difficult in this disease as different endpoints are relevant for different manifestations of the disease. We propose that targeting the underlying determinants of specific characteristics of patients with bronchiectasis will be more likely to yield therapeutic advances that improve QoL and outcomes.
